# Supplementary material for: An Engineered Hybrid Protein from Dermatophagoides pteronyssinus Allergens Shows Hypoallergenicity
Source: Int J Mol Sci. 2019 Jun 21;20(12):3025. doi: 10.3390/ijms20123025 (PMC6628193; doi:10.3390/ijms20123025)
Supplement: Supplementary file 1 [file ijms-20-03025-s001.pdf]

SUPPLEMENTARY MATERIAL

Table 1. Secondary structure calculations for DPx4 are based on the CD-data. For both the homology model of DPx4 and crystal structure of Der p 1 (PDB-Code: 3F5V) values were calculated with PDBsum (http://www.ebi.ac.uk/pdbsum). Secondary structure contents are given in percentages (%) of α-helix, β-sheet and others.

Secondary Structure content	α-helix	β-sheet	Others
*DPx4 (CD-curve)**	10%	39%	51%
*Der p 1***	25%	21%	54%
*Homology model***	23%	13%	64%

*   Calculated with Dichroweb

** Calculated with PDBsum - EMBL-EBI

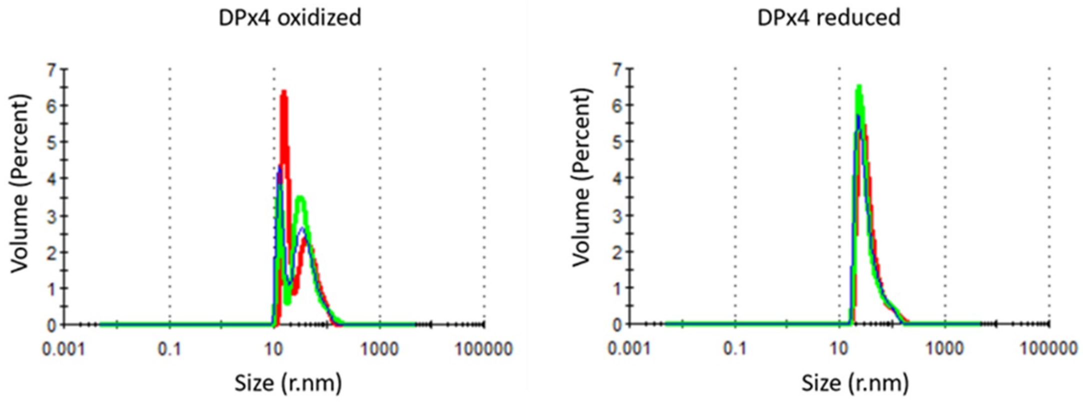

Peak	Size (nm)	Mass (kDa)	%Pd
1	17.01 ± 2.699	$2.55x10^{3}$ ± 996,2	15.3
2	62.03 ± 23.37	$5,27x10^{4}$ ± $9,01x10^{3}$	35.9

Peak	Size (nm)	Mass (kDa)	%Pd
1	40.93 ± 34.33	$1.99x10^{4}$ ± $2.9x10^{4}$	55.4

Figure S1: DLS analysis of DPx4. The figure shows the size distribution in r.nm by mass of DPx4 in an oxidized form (left panel) and reduced form (right panel) with three repeat measurements each. The size in r.nm, the estimated MW in kDa and the polydispersity (Pd) in % of the single peaks is listed below each panel.

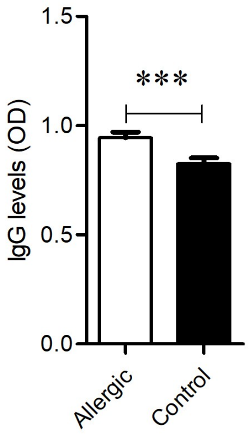

Figure S2: Serum IgG levels to DPx4 by ELISA. IgG levels to hybrid protein in 90 serum samples from allergic and 55 non-allergic individuals.

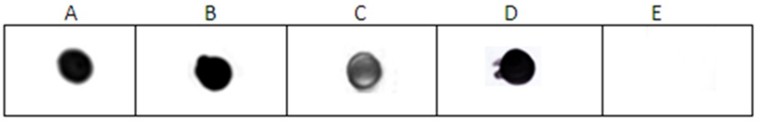

Figure S3: Dot-blot assay with monoclonal antibodies. Hybrid protein, Der p 1, Der p 2 and BSA were dotted onto nitrocellulose membrane and incubated with monoclonal antibodies as indicated (Protein/antibody): A) Der p 1 / Anti Der p 1, B) Der p 2 /Anti Der p 2, C) DPx4/ Anti-Der p 1, D) DPx4 /Anti – Der p 2, E) BSA/ Anti Der p 1. Anti-Der p 1 monoclonal antibody (4C1, Indoor Biotechnologies). Anti-Der p 2 monoclonal antibody (Dpx-A9, Indoor Biotechnologies).

```
      Tag
  1 MAHHHHHHVT EPCIIHRGKP FEFEVRQFAN VTPIRMQGGC GSCWAFSGVA
 51 ATEAYLQLIE EDLERSEERL VDCASQHNGC HGDTIPRGIE YIQHNGVVQE
101 SYYRYVAREQ QSCRRPNAQR FYDIKYTWNV PKIAPKSVNK IREALAQTHS
151 AIAVIIGIKD LDAFRHYDGR TIIQRDNGYQ PNYHAVNIVG YSNAQGVDYW
201 IVRNSWDTNW GDNGYGYFAV LSDVLTAEEY PYVVIL
```

Figure S4. Amino acid sequence of DPx4.
